# Naturally occurring recombination in ferret coronaviruses revealed by complete genome characterization

**DOI:** 10.1099/jgv.0.000520

**Published:** 2016-09

**Authors:** Mart M. Lamers, Saskia L. Smits, Gadissa B. Hundie, Lisette B. Provacia, Marion Koopmans, Albert D. M. E. Osterhaus, Bart L. Haagmans, V. Stalin Raj

**Affiliations:** ^1^​ Department of Viroscience, Erasmus MC, Rotterdam, The Netherlands; ^2^​ Artemis One Health, Utrecht, The Netherlands; ^3^​ Center for Infection Medicine and Zoonoses Research, University of Veterinary Medicine, Hannover, Germany

**Keywords:** ferret coronavirus, alphacoronavirus, recombination, complete genome

## Abstract

Ferret coronaviruses (FRCoVs) exist as an enteric and a systemic pathotype, of which the latter is highly lethal to ferrets. To our knowledge, this study provides the first full genome sequence of a FRCoV, tentatively called FRCoV-NL-2010, which was detected in 2010 in ferrets in The Netherlands. Phylogenetic analysis showed that FRCoV-NL-2010 is most closely related to mink CoV, forming a separate clade of mustelid alphacoronavirus that split off early from other alphacoronaviruses. Based on sequence homology of the complete genome, we propose that these mustelid coronaviruses may be assigned to a new species. Comparison of FRCoV-NL-2010 with the partially sequenced ferret systemic coronavirus MSU-1 and ferret enteric coronavirus MSU-2 revealed that recombination in the spike, 3c and envelope genes occurred between different FRCoVs.

Coronaviruses (CoVs) are large enveloped, positive-stranded RNA viruses classified under the subfamily *Coronavirinae* within the family *Coronaviridae*, order Nidovirales. They are subdivided into four genera, *Alphacoronavirus, Betacoronavirus, Gammacoronavirus* and *Deltacoronavirus*. CoVs infect birds and mammals, including humans, and are known for their ability to jump the species barrier, which may be facilitated by their high mutation and recombination rates. This is exemplified by the betacoronavirus severe acute respiratory syndrome CoV (SARS-CoV), which emerged in 2002 from bats in the Guangdong province of China and subsequently spread to 29 countries, resulting in more than 8000 cases with at least 700 fatalities (Drosten *et al.*, 2003; Ksiazek *et al.*, 2003; Peiris *et al.*, 2003). Approximately 10 years later, a second highly pathogenic human betacoronavirus, Middle East respiratory syndrome CoV (MERS-CoV), was discovered in the Middle East as a zoonotic pathogen from dromedary camels, and is continuing to cause human infections (Zaki *et al.*, 2012; Haagmans *et al.*, 2014; Reusken *et al.*, 2013; Raj *et al.*, 2014).

Alphacoronaviruses include viruses that cause common cold in humans (HCoV-229E and HCoV-NL63) and some important causes of enteric disease in domestic animals, including transmissible gastroenteritis virus of swine (TGEV), porcine epidemic diarrhoea virus (PEDV), canine CoV and feline coronavirus (FCoV). Recently, a novel ferret enteric CoV (FRECV) was identified in domesticated ferrets (*Mustela putorius*) in which epizootic catarrhal enteritis had been diagnosed; the illness was characterized by foul-smelling green diarrhoea with high mucous content, lethargy, anorexia and vomiting, but not by high mortality rates (Wise *et al.*, 2006). A closely related, but fatal ferret CoV, ferret systemic coronavirus (FRSCV) was detected in ferrets with systemic pyogranulomatous inflammation (Garner *et al.*, 2008; Wise *et al.*, 2010; Martínez *et al.*, 2008). This disease strongly resembled the clinical and pathologic features of the dry form of feline infectious peritonitis, which is associated with a FCoV, feline infectious peritonitis virus (FIPV). By comparisons of genomic sequences of different FCoVs it was found that the severe pathotype of FIPV may arise through mutations in the spike and in the 3c genes of the less pathogenic feline enteric CoV (Pedersen, 2009; Chang *et al.*, 2010; Dewerchin *et al.*, 2005; Licitra *et al.*, 2013; Rottier *et al.*, 2005). Similarly, Wise *et al*. (2010) have shown that FRECV and FRSCV differ significantly in spike protein and that deletions in FRCoV 3c may also correlate with the severe pathotype of FRSCV. Despite the fact that genetic comparisons of FRCoVs may provide significant insights into how these and other CoVs are able to alter their tropism and pathogenicity, no full genome sequences of FRCoVs are available to date.

In 2010, we investigated the prevalence of FRCoVs in asymptomatic ferrets from ferret farms in The Netherlands, and found that ~60 % of the samples were PCR positive for a FRCoV (Provacia *et al.*, 2011). Interestingly, sequence analysis of the partial spike gene clearly showed that this enteric virus was more closely related to the FRSCV strain MSU-1 than to FRECV strain MSU-2 (hereafter referred to as FRSCV and FRECV, respectively). In this study, we selected one rectal swab sample with high viral load (cycle threshold 16.3) from the 2010 ferret sample collection for further genomic analysis. RNA was isolated from 140 µl of rectal swab sample in viral transport medium (VTM) with the QIAamp Viral RNA Mini kit (Qiagen). Next, the sample was subjected to full genome sequencing using 454 deep-sequencing (454 GS Junior Instrument; Roche) as described elsewhere (Allander *et al.*, 2001, 2005; van den Brand *et al.*, 2011; van Leeuwen *et al.*, 2010). A total of 223 107 sequence reads were obtained and sequences were trimmed and assembled using the *de novo* assembly module in CLC Genomics Workbench 4.5.1 (CLC Bio) (Losada *et al.*, 2011). blastx (Altschul *et al.*, 1997) analysis of obtained reads revealed sequences that were most closely related to FRSCV and FRECV. *De novo* assembly revealed the consensus sequence of FRCoV, tentatively called FRCoV-NL-2010. In total, 25 313 reads were specific for FRCoV-NL-2010, revealing 99.73 % of the genome with a coverage ranging from 1 to 2260 reads at single nucleotide positions. Gaps or regions with coverage of <4 reads were confirmed by Sanger sequencing. Using 5′ and 3′ rapid amplification of cDNA ends (RACE), the ends of the genome were obtained, leading to a complete genome sequence consisting of 28 434 nucleotides, including 18 nucleotides of the poly-A tail (Genbank accession number KM347965). After genome assembly, the raw sequence reads were mapped against the complete FRCoV-NL-2010 genome, which revealed that only one CoV was contained in the sample.

CoVs contain the largest genomes among RNA viruses, ranging from 27 to 31 kb (Gorbalenya *et al.*, 2006). The genomes consist of polycistronic positive-stranded RNA and contain two large 5′-proximal replicase ORFs, ORF1a and ORF1b, which occupy three-quarters of the genome. These are translated to produce polyprotein 1a (pp1a) and, following −1 ribosomal frameshifting, polyprotein 1ab (pp1ab). These polyproteins are cleaved into 15 or 16 nonstructural proteins (NSPs) (Gorbalenya *et al.*, 2006; Ziebuhr *et al.*, 2000). Ribosomal frameshifting is thought to be mediated by a slippery sequence 5′-ACAACT-3′ that is conserved across all CoVs. The region downstream of ORF1b contains a number of smaller ORFs that are transcribed as subgenomic mRNAs. These mRNAs are composed of a common 5′ leader and a variable part consisting of at least one ORF. The common leader sequence is joined during discontinuous negative-strand RNA synthesis in a process that is directed by base-pairing interactions between conserved transcription-regulatory sequences (TRSs). TRSs are found at the 3′ end of the leader sequence (leader TRS) and upstream of most 3′ subgenomic ORFs (body TRSs).

Analysis of the complete genome of FRCoV-NL-2010 revealed the two large ORFs, ORF1a and ORF1b, as well as at least seven ORFs, ORF2–8, at the 3′ end of the genome (Fig. 1a, Table S1, available in the online Supplementary Material). According to sequence conservation analyses performed with other CoVs ORF2, -4, -5 and -6 are predicted to encode the four structural proteins of CoVs, spike (S), envelope (E), membrane (M) and nucleocapsid (N), respectively. ORF3, -7 and -8 were homologous to the 3c, 3x and 7b genes of FCoVs, respectively. A sequence identical to the conserved CoV slippery site was found in the overlapping region of ORF1a and ORF1b. Putative NSP functional domains were predicted using ZCURVE_CoV 2.0 (Gao *et al.*, 2003) and sequence comparison of FRCoV-NL-2010 with other alphacoronaviruses allowed the prediction of the putative pp1a and pp1ab cleavage sites and annotation of the 15 NSPs found in alphacoronaviruses (Table S2). A leader TRS and five putative body TRSs could be identified in the genome, with the sequence 5′-CTAAAC-3′ forming the TRS core (Fig. 1b). Experimental studies are needed to confirm the correct identification of the TRSs in the FRCoV-NL-2010 genome.

**Fig. 1.**
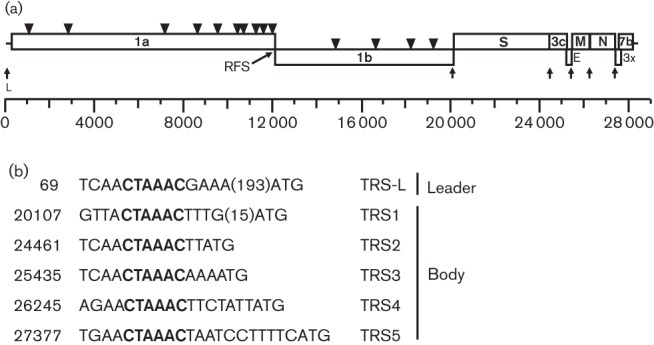
Genome organization of FRCoV-NL-2010. (a) Genome organization of FRCoV-NL-2010. Triangles indicate predicted cleavage sites, arrows indicate trailer and leader TRSs. RFS, ribosomal frame-shifting sequence. (b) Putative leader and body TRSs in the genome of FRCoV-NL-2010. The core TRS is indicated in bold.

Pairwise comparison of FRCoV-NL-2010 with other partially sequenced FRCoVs revealed that it contained an intact ORF3c, which would be expected on the basis of its enteric pathotype. For FIPV, it is thought that the loss of 3c gene function enhances the internalization and replication of FIPV in macrophages, which would aid in spreading the virus systemically (Dewerchin *et al.*, 2005). In accordance, partial genome sequencing by Wise *et al*. (2010) revealed that two out of three FRSCV strains contained deletions in 3c, while the two FRECV strains that were included in the study contained an intact 3c.

Phylogenetic analysis of the full genome and ORF1ab confirmed that this virus belongs to the genus *Alphacoronavirus* and clusters most closely with mink CoV, forming a separate clade of mustelid alphacoronavirus that clusters intermediately between the *Alphacoronavirus 1 *species and other alphacoronaviruses (Fig. 2a, b). FRCoV and mink CoVs share 69.2–69.7 % nucleotide identity with members of the *Alphacoronavirus 1* species, which is less than members of this species share with one another (>88.6 %), supporting the suggestion of Vlasova *et al*. (2011) that mustelid alphacoronaviruses should be assigned to a separate species. This proposed designation as a new species is also supported by the International Committee on Taxonomy of Viruses (de Groot *et al.*, 2012) species demarcation criteria that the minimum amino acid identity within each genus needs to be 90 % in seven conserved replicase domains (ADRP, 3CLpro, RdRP, Hel, ExoN, NendoU and O-MT). The amino acid identity between FRCoV-NL-2010 and the mink CoVs was 90 % in these seven conserved replicase domains. While members of the *Alphacoronavirus 1* species share >96 % amino acid identity in these regions, FRCoV-NL-2010 and the mink CoVs displayed a maximum amino acid identity of 82 % with members of *Alphacoronavirus 1 *and other alphacoronaviruses in these regions. Consequently, FRCoV-NL-2010 and mink CoVs cannot be assigned to the *Alphacoronavirus 1 *species. These viruses may therefore be designated a new species, tentatively named *Alphacoronavirus 2*, which should probably also include FRECV and FRSCV based on available partial sequence information.

**Fig. 2.**
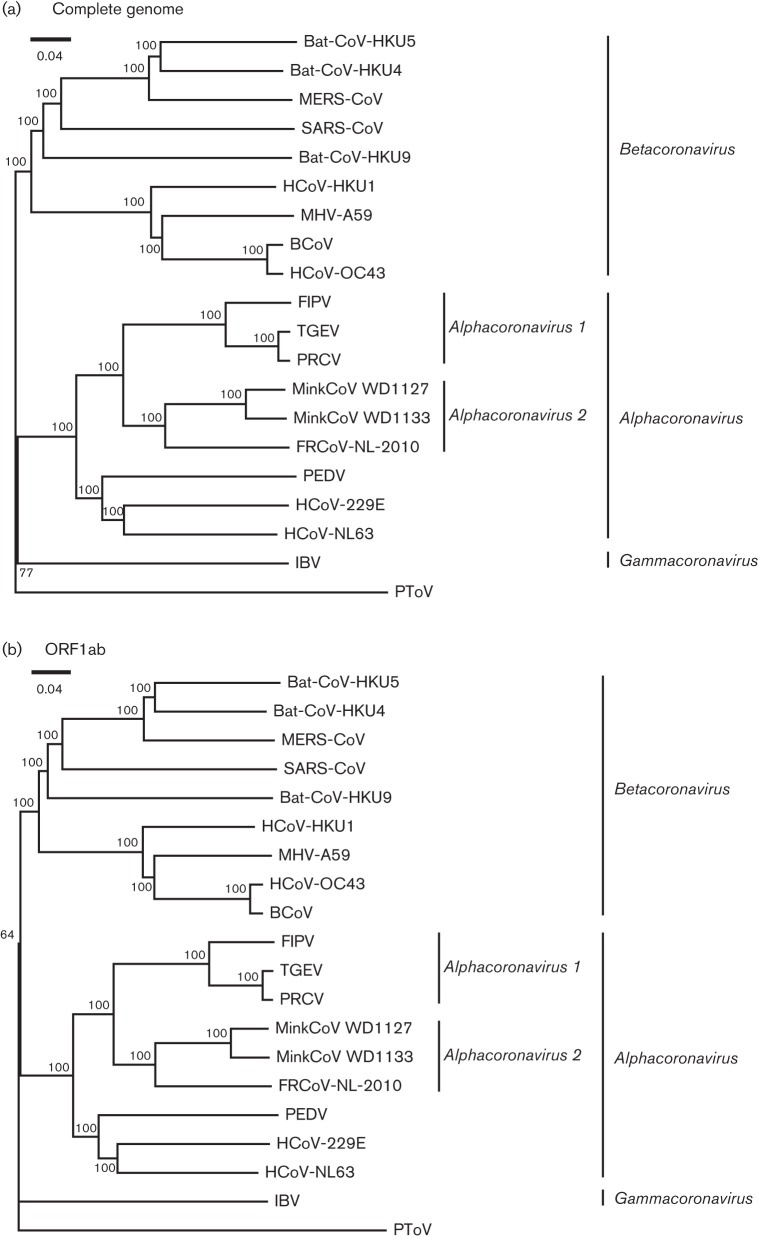
Phylogenetic analysis of FRCoV-NL-2010. Neighbour-joining trees of the complete genome (a) and ORF1ab (b) of selected isolates, aligned by ClustalW, were inferred by the *p*-distance model in mega5 (http://www.megasoftware.net). Indicated bootstrap values at nodes were calculated on 1000 replicates. Scale bars indicate nucleotide substitutions per site. BCoV, bovine coronavirus; IBV, infectious bronchitis virus; HCoV, human coronavirus; MHV, mouse hepatitis virus; PRCV, porcine respiratory coronavirus; PToV, porcine torovirus; see text for other abbreviations. Accession numbers: HCoV-229E (NC002645); HCoV-NL63 (NC005831); PEDV (NC003436); FIPV (NC002306); TGEV (DQ811785); HCoV-OC43 (AY391777); BCoV (BCU00735); SARS-CoV (NC004718); MinkCoV WD1127 (HM245925); MinkCoV WD1133 (HM245926); MHV-A59 (NC001846); HCoV-HKU1 (NC006577); Bat-CoV-HKU4 (NC009019); Bat-CoV-HKU5 (NC009020); PRCV (DQ811787); PToV (NC022787); FRCoV-NL-2010 (KM347965); MERS-CoV (JX869059); IBV (NC001451).

Although there are no full genomic sequences available of other FRCoVs, a phylogenetic tree of the partial N gene shows a close relationship of FRCoV-NL-2010 with the canonical FRCoVs FRSCV and FRECV, as well as with the enteric FRCoVs 511c and 4E98 that were found in The Netherlands in 2010 (Provacia *et al.*, 2011) (Fig. 3a). Phylogenetic analysis of a small fragment of the S gene indicates that in this region the virus is most closely related to the viruses found in The Netherlands. This tree also shows that in this region, lying 3′ proximal in the S gene, FRCoV-NL-2010 is more related to FRSCV than to FRECV (Fig. 3a), whereas this trend was not observed for the N gene, suggesting a recombination event. Next, recombination events were analysed using the 3′-most ~8 kb sequence which is sequenced for both FRSCV and FRECV. Six methods, comprising recombination detection program (RDP), geneconv, Bootscan, Maxchi, Chimaera and 3seq, implemented in RDP4 (Martin *et al.*, 2015) were used, with likely parental isolates and recombination breakpoints determined using default settings. A recombination event was detected for the region between 1718 and 5432 (numbering based on alignment) using the indicated methods (*P*-values ranged from 1.110×10^−16^ to 2.236×10^−81^). The Bootscan output is shown in Fig. 3b. This region includes two-thirds of the S gene, the entire 3c gene and part of the E gene. In the region before the recombination breakpoint (corresponding to ORF1b and the first third of the S gene; S1–S1412, FRCoV-NL-2010 numbering), FRCoV-NL-2010 was an outgroup of FRECV and FRSCV. Inside the recombination region, two phylogenetic groupings can be observed (Fig. 3c). In two-thirds of the S gene (S1412–S4239, FRCoV-NL-2010 numbering), FRCoV-NL-2010 clustered with FRSCV; while in a short 3′ proximal part of the S gene and the majority of the 3c gene (S4240–3c471) FRCoV-NL-2010 grouped with FRECV. This indicated that there was a second recombination event lying within the recombination region. Indeed, in this region a recombination event was found ranging from position 4534 to 5369 in the alignment. This event was detected by the same six methods with *P*-values ranging from 2.416×10^−02^ to –3.356×10^−17^. On the basis of these findings, we propose an evolutionary pathway in which the ancestor of FRCoV-NL-2010 donated two-thirds of its S gene, its 3c gene and part of the E gene to a FRECV-like virus (Fig. 3d). The result of this recombination event may have been the ancestor of FRSCV if this virus later acquired deletions in the 3c and 3x genes. The second recombination event may have been between a FRECV-like virus and the ancestor of FRCoV-NL-2010, in which the former donated its 3c gene. Additional sequence information is needed in order to support these hypotheses.

**Fig. 3.**
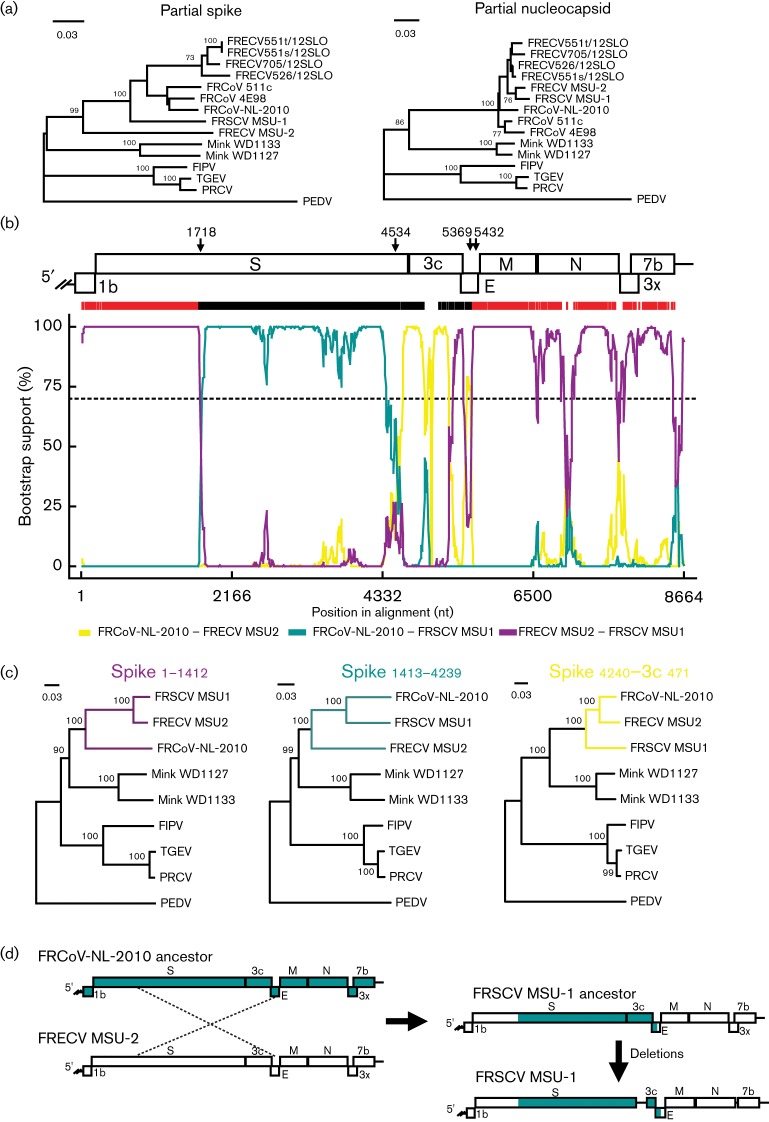
Recombination analysis of available ferret coronavirus (FRCoV) genome fragments. (a) Neighbour-joining trees of a small region in the spike gene (nucleotide position 3598–4089) and nucleocapsid (nucleotide position 61–312). (b) Recombination analysis by Bootscan, implemented in RDP4 (Martin *et al.*, 2015), with recombination break-points determined using default settings. Break-points (indicated with arrows) of the first and second recombination event were detected at nucleotide positions 1718 and 5432, and 4534 and 5369 (based on the position in the alignment), respectively. Window size was set at 200 and the step size at 10. The dashed line indicates the bootstrap threshold of 70 %. (c) Neighbour-joining trees of nucleotides 1–1412 and 1413–4239 of the FRCoV-NL-2010 S gene, and nucleotides 4240 of the S gene until 471 in the 3c gene. Selected isolates were aligned by clustalw and trees were inferred by the *p*-distance model in mega5 (http://www.megasoftware.net). Indicated bootstrap values at nodes were calculated on 1000 replicates. Scale bars in (a) and (c) indicate nucleotide substitutions per site. Hypothetical evolution of FRSCV MSU-1, FRECV MSU-2 and FRCoV-NL-2010 in which the ancestor of FRCoV-NL-2010 donated two-thirds of its S gene, its 3c gene and part of its E gene to FRECV, generating the ancestor of FRSCV which subsequently acquired deletions in the 3c and 3x genes (d).

This study underlines that CoVs can exchange genes that are likely to be major determinants of pathogenicity. Therefore, recombination may be a driving force for the formation of pathogenic viruses from less pathogenic viruses. However, this study does not provide evidence that recombination can directly alter the pathotype of a virus, as the backbones of the investigated viruses differ substantially. Mutations or deletions in the 3c gene are likely to play a major role. Another limitation of this study is that the recombination analysis only involved the 3′ third of the genome as the genomes of FRECV and FRSCV have not been sequenced completely. To further characterize the determinants of pathogenicity of FRCoVs, more sequence information is needed and, importantly, these viruses will need to be isolated or rescued using reverse genetic techniques in order to perform experimental inoculation of ferrets.

In conclusion, to our knowledge this study provides the first full genome of a FRCoV, which was detected in The Netherlands in 2010 in ferrets. We show that it separates phylogenetically in a clade with other FRCoVs and mink CoVs. This clade meets the ICTV criteria for species demarcation. In addition, the new sequence information provided by the full genome of FRCoV-NL-2010 allows the identification of recombination among FRCoVs in the S, 3c and E genes.

## Supplementary Data

39 Supplementary File 1

## References

[R1] Allander T., Emerson S. U., Engle R. E., Purcell R. H., Bukh J. (2001). A virus discovery method incorporating DNase treatment and its application to the identification of two bovine parvovirus species. Proc Natl Acad Sci U S A.

[R2] Allander T., Tammi M. T., Eriksson M., Bjerkner A., Tiveljung-Lindell A., Andersson B. (2005). Cloning of a human parvovirus by molecular screening of respiratory tract samples. Proc Natl Acad Sci U S A.

[R3] Altschul S. F., Madden T. L., Schäffer A. A., Zhang J., Zhang Z., Miller W., Lipman D. J. (1997). Gapped BLAST and PSI-BLAST: a new generation of protein database search programs. Nucleic Acids Res.

[R4] Chang H. W., de Groot R. J., Egberink H. F., Rottier P. J. (2010). Feline infectious peritonitis: insights into feline coronavirus pathobiogenesis and epidemiology based on genetic analysis of the viral 3c gene. J Gen Virol.

[R30] de Groot R. J., Baker S. C., Baric R., Enjuanes L., Gorbalenya A. E., Holmes K., Perlman S., Poon L., Rottier P. J. M., other authors, King A. M. Q., Adams M. J., Cartens E. B., Lefkowitz. E. J. (2012). Virus Taxonomy, the 9th Report of the International Committee on Taxonomy of Viruses. Family Coronaviridae.

[R5] Dewerchin H. L., Cornelissen E., Nauwynck H. J. (2005). Replication of feline coronaviruses in peripheral blood monocytes. Arch Virol.

[R6] Drosten C., Günther S., Preiser W., van der Werf S., Brodt H. R., Becker S., Rabenau H., Panning M., Kolesnikova L. (2003). Identification of a novel coronavirus in patients with severe acute respiratory syndrome. N Engl J Med.

[R7] Gao F., Ou H. Y., Chen L. L., Zheng W. X., Zhang C. T. (2003). Prediction of proteinase cleavage sites in polyproteins of coronaviruses and its applications in analyzing SARS-CoV genomes. FEBS Lett.

[R8] Garner M. M., Ramsell K., Morera N., Juan-Sallés C., Jiménez J., Ardiaca M., Montesinos A., Teifke J. P., Löhr C. V. (2008). Clinicopathologic features of a systemic coronavirus-associated disease resembling feline infectious peritonitis in the domestic ferret (*Mustela putorius*). Vet Pathol.

[R9] Gorbalenya A. E., Enjuanes L., Ziebuhr J., Snijder E. J. (2006). Nidovirales: evolving the largest RNA virus genome. Virus Res.

[R10] Haagmans B. L., Al Dhahiry S. H., Reusken C. B., Raj V. S., Galiano M., Myers R., Godeke G. J., Jonges M., Farag E. (2014). Middle East respiratory syndrome coronavirus in dromedary camels: an outbreak investigation. Lancet Infect Dis.

[R11] Ksiazek T. G., Erdman D., Goldsmith C. S., Zaki S. R., Peret T., Emery S., Tong S., Urbani C., Comer J. A. (2003). A novel coronavirus associated with severe acute respiratory syndrome. N Engl J Med.

[R12] Licitra B. N., Millet J. K., Regan A. D., Hamilton B. S., Rinaldi V. D., Duhamel G. E., Whittaker G. R. (2013). Mutation in spike protein cleavage site and pathogenesis of feline coronavirus. Emerg Infect Dis.

[R13] Losada L., Varga J. J., Hostetler J., Radune D., Kim M., Durkin S., Schneewind O., Nierman W. C. (2011). Genome sequencing and analysis of *Yersina pestis* KIM D27, an avirulent strain exempt from select agent regulation. PLoS One.

[R14] Martin D. P., Murrell B., Golden M., Khoosal A., Muhire B. (2015). RDP4: detection and analysis of recombination patterns in virus genomes. Virus Evolution.

[R15] Martínez J., Reinacher M., Perpiñán D., Ramis A. (2008). Identification of group 1 coronavirus antigen in multisystemic granulomatous lesions in ferrets (*Mustela putorius furo*). J Comp Pathol.

[R16] Pedersen N. C., Liu H., Dodd K. A., Pesavento P. A. (2009). Significance of coronavirus mutants in feces and diseased tissues of cats suffering from feline infectious peritonitis. Viruses.

[R17] Peiris J. S., Lai S. T., Poon L. L., Guan Y., Yam L. Y., Lim W., Nicholls J., Yee W. K., Yan W. W. (2003). Coronavirus as a possible cause of severe acute respiratory syndrome. Lancet.

[R18] Provacia L. B., Smits S. L., Martina B. E., Raj V. S., Doel P. V., Amerongen G. V., Moorman-Roest H., Osterhaus A. D., Haagmans B. L. (2011). Enteric coronavirus in ferrets, The Netherlands. Emerg Infect Dis.

[R19] Raj V. S., Farag E. A., Reusken C. B., Lamers M. M., Pas S. D., Voermans J., Smits S. L., Osterhaus A. D., Al-Mawlawi N. (2014). Isolation of MERS coronavirus from a dromedary camel, Qatar, 2014. Emerg Infect Dis.

[R20] Reusken C. B., Ababneh M., Raj V. S., Meyer B., Eljarah A., Abutarbush S., Godeke G. J., Bestebroer T. M., Zutt I. (2013). Middle East Respiratory Syndrome coronavirus (MERS-CoV) serology in major livestock species in an affected region in Jordan, June to September 2013. Euro Surveill.

[R21] Rottier P. J., Nakamura K., Schellen P., Volders H., Haijema B. J. (2005). Acquisition of macrophage tropism during the pathogenesis of feline infectious peritonitis is determined by mutations in the feline coronavirus spike protein. J Virol.

[R22] van Boheemen S., de Graaf M., Lauber C., Bestebroer T. M., Raj V. S., Zaki A. M., Osterhaus A. D., Haagmans B. L., Gorbalenya A. E. (2012). Genomic characterization of a newly discovered coronavirus associated with acute respiratory distress syndrome in humans. MBio.

[R24] van den Brand J. M., van Leeuwen M., Schapendonk C. M., Simon J. H., Haagmans B. L., Osterhaus A. D., Smits S. L. (2011). Metagenomic analysis of the viral flora of pine marten and European badger feces. J Virol.

[R23] van Leeuwen M., Williams M. M., Koraka P., Simon J. H., Smits S. L., Osterhaus A. D. (2010). Human picobirnaviruses identified by molecular screening of diarrhea samples. J Clin Microbiol.

[R25] Vlasova A. N., Halpin R., Wang S., Ghedin E., Spiro D. J., Saif L. J. (2011). Molecular characterization of a new species in the genus *Alphacoronavirus* associated with mink epizootic catarrhal gastroenteritis. J Gen Virol.

[R26] Wise A. G., Kiupel M., Maes R. K. (2006). Molecular characterization of a novel coronavirus associated with epizootic catarrhal enteritis (ECE) in ferrets. Virology.

[R27] Wise A. G., Kiupel M., Garner M. M., Clark A. K., Maes R. K. (2010). Comparative sequence analysis of the distal one-third of the genomes of a systemic and an enteric ferret coronavirus. Virus Res.

[R28] Zaki A. M., van Boheemen S., Bestebroer T. M., Osterhaus A. D., Fouchier R. A. (2012). Isolation of a novel coronavirus from a man with pneumonia in Saudi Arabia. N Engl J Med.

[R29] Ziebuhr J., Snijder E. J., Gorbalenya A. E. (2000). Virus-encoded proteinases and proteolytic processing in the *Nidovirales*. J Gen Virol.

