# Supplementary material for: Naturally occurring recombination in ferret coronaviruses revealed by complete genome characterization
Source: J Gen Virol. 2016 Sep;97(9):2180–6. doi: 10.1099/jgv.0.000520 (PMC7079585; doi:10.1099/jgv.0.000520)
Supplement: Supplementary file 1 [file jgv-97-2180-s001.pdf]

Supplementary table 1: Open reading frames and corresponding genomic positions of FRCoV-NL-2010.

Open reading frame	Genomic position (nt)	Protein product
ORF1a/ab	276..12122, 12122..20143	Polyprotein 1a/ab
ORF2	20136…24452	Spike
ORF3	24473…25216	3c
ORF4	25185…25433	Envelope
ORF5	25448…26245	Membrane
ORF6	26262…27389	Nucleocapsid
ORF7	27398…27670	3x
ORF8	27561…28169	7b

Abbreviations: ORF, open reading frame; nt, nucleotide.

Supplementary table 2: Cleavage products of the replicase polyproteins of FRCoV-NL-2010

Cleavage product	Position in polyprotein pp1a/pp1ab	Protein size (no.of aminoacids)	Putative functional domain
nsp1	1Met-Gly289	289	
nsp2	290Asn-Gly876	587	
nsp3	877Gly-Gly2326	1450	ADRP, PLpro
nsp4	2327Ser-Gln2818	492	
nsp5	2819Ser -Gln3120	302	3CLpro
nsp6	3121Ser-Gln3415	295	
nsp7	3416Ser-Gln3498	83	
nsp8	3499Ser-Gln3693	195	
nsp9	3694Asn-Gln3804	111	
nsp10	3805Ala-Gln3939	135	
nsp12	3940Ala-Gln4868	929	RdRp
nsp13	4869Ala-Gln5465	597	HEL1
nsp14	5466Ser-Gln5984	519	ExoN
nsp15	5985Ser-Gln6323	339	NendoU
nsp16	6324Ser-Val6622	299	O-MT

Abbreviations: ADRP, ADP-ribose 1_-phosphatase; PLpro, papain-like proteinase ; 3CLpro, 3C-like cysteine proteinase; RdRp, RNA-dependent RNA polymerase; HEL1, superfamily 1 helicase; ExoN, 3-to-5 exonuclease; NendoU, nidoviral endoribonuclease specific for U; O-MT, S-adenosylmethionine-dependent ribose 2-O-methyltransferase.
